# Practical tips to improve bedside teaching using learning theories and critical reasoning

**DOI:** 10.12688/mep.19826.1

**Published:** 2023-10-12

**Authors:** Thomas Rotthoff

**Affiliations:** 1 Medical Didactics and Education Research (DEMEDA), Augsburg University, Augsburg, 86259, Germany

**Keywords:** bedside teaching, improve bedside teaching, medical education bedside teaching

## Abstract

Bedside teaching offers the opportunity to integrate the different professional roles and competencies of doctors and medical students with one another. It should not be delivered uniformly to all students but must be adapted to the level of experience of the students. Students at an early stage of their studies need a greater degree of structure and scaffolding than advanced students, as they may still feel insecure regarding a variety of factors. It therefore seems useful to take a closer look at the cognitive theories behind bedside teaching while bearing in mind that, in comparison to other teaching and learning formats, findings about emotion, epistemic beliefs, visual thinking strategies, theories of cognitive load, experiential learning and scripting, critical reasoning, structured briefing and debriefing can improve bedside teaching. This paper provides practical tips to reveal the processes of clinical reasoning and decision-making in a more rational, structured, analytical and critical manner.

## Introduction

Unlike other teaching formats, bedside teaching is authentic and offers the opportunity to integrate the different professional roles and competencies of doctors and medical students with one another. Bedside teaching is the only format in medical education in which the skills of history-taking and physician–patient communication, physical examination, clinical reasoning, decision-making, empathy and professionalism can be simultaneously taught and learned as an integrated entity in a real clinical setting ( Garout *et al.*, 2016; Spencer, 2003). However, this approach seems to be increasingly under pressure today ( Rotthoff, 2022). For competency-based medical education, bedside teaching is required to promote, monitor and assess students’ continuous development in competence and performance. Theories suggest the value of involving patients at an early stage of a student’s medical education ( Dornan *et al.*, 2006; Wenrich *et al.*, 2013; Yardley *et al.*, 2013) because early experiences with patients can help students socialise to the medical profession and strengthen their learning and skills acquisition ( Dornan *et al.*, 2006). Accordingly, bedside teaching should not be delivered uniformly to all students but must be adapted to the level of experience of the students. Students at an early stage of their studies need a greater degree of structure and scaffolding during bedside teaching than advanced students, as they may still feel insecure regarding a variety of factors. For example, they may have insecurities about their professional skills, a growing realisation that medicine is often inexact or a heightened awareness of the responsibility associated with patient care ( Evans *et al.*, 2012; Nevalainen *et al.*, 2010). Given this context, bedside teaching is a frequently underestimated and demanding instructional method that transcends the role of merely practising medicine in the presence of students.

It is therefore important to take a closer look at the cognitive theories behind bedside teaching while bearing in mind that, in comparison to other teaching and learning formats, findings about emotion, epistemic beliefs, visual thinking strategies, theories of cognitive load, experiential learning and scripting, critical reasoning, structured briefing and debriefing can improve bedside teaching. These assumptions provide the basis for the eleven tips that follow.

***Tip 1***: *Consider the influences of emotion on learning*

For students in the first semesters of their studies, the clinical routine is often not familiar, and the patients’ conditions, medical histories and similar may trigger emotions that have not yet been processed professionally. In a performance situation, a person needs to integrate their cognitive and affective resources and skills dynamically. Excitement and emotion can influence learning by affecting students’ attention, motivation and self-regulation. Situations that trigger anxiety and shame may result in the adoption of inflexible learning approaches, such as mere repetition and rote memorisation ( Pekrun, 2014), which can limit students’ performance and learning. However, bedside teaching also provides a unique chance to foster learning by triggering emotions. Abundant evidence has firmly established that emotional events are recalled with greater clarity, accuracy, and longevity compared to neutral events ( Tyng *et al.*, 2017).

- Take into account that inexperienced students can become overwhelmed in a clinical situation. Prepare students for the situation via a structured briefing (see below).
- Start with the students’ existing competence and skills levels to help them regulate their emotions. Activate the students’ prior knowledge.
- Support the students by naming and interpreting stressors so that they result in eustress rather than distress ( Jamieson *et al.*, 2013; Rudland *et al.*, 2020).
- Reduce extraneous stressors (e.g. personal pressure to do well) and promote stressors that are more likely to facilitate learning and cognitive performance by, for example, reducing the complexity of the situation.
- Arouse the curiosity of your students to stimulate learning ( Tyng *et al.*, 2017).
- Keep in mind that students have different mindsets, personality traits and self-beliefs regarding how to react in challenging situations.

***Tip 2***: *Start with a briefing*

Extraneous stressors could be reduced by briefing students. Briefing serves to focus attention in a targeted manner and involves interaction between the teacher and students immediately prior to the learning experience. Some research is available on the effectiveness of briefing in simulated settings, but the volumes are considerably less compared to debriefing ( Tyerman *et al.*, 2019). Transferability to a real clinical context is to be assumed. The briefing takes place in close proximity to the patient, typically in front of the patient room.

- Reduce the briefing information to the essentials in order to prepare the students for situations where they will require their working memory but at the same time not overload it.
- Provide students with just the essential information about the patient and the setting: What can the students expect to see and experience behind the door? What kind of patient will they see, and what is the patient’s mental and physical condition?
- Address the learning objectives.
- What do you expect from your students? Do they have to perform a task or demonstrate something on the patient? In what manner? What will the students’ specific roles be?
- Give advice on special instructions (e.g. hygiene or even issues to be avoided).
- Move more detailed information about the patient or pathophysiological explanations to the encounter, debriefing or detached teaching sessions.

***Tip 3***: *Reduce the cognitive load*

Students can be overwhelmed in situations that you as a clinician process automatically due to your clinical expertise. Supposedly simple cases can be complex for students. The greater your prior knowledge and familiarity with an environment, the better your working memory can draw on information stored in your long-term memory, which is already organised. Working memory has strong capacity limits, which is a limitation in the cognitive processing of new information. Working memory is responsible for actively processing and handling information in real time and receives inputs from both sensory systems (vision, touch, hearing) and long-term memory. Overload can occur when students are confronted with environments and dynamic visual and emotional situations they are not familiar with (e.g. patients with many catheters or monitoring systems, abnormal physical findings) and distractions, such as other patients in the room or family members.

If students have only little prior knowledge or experience, they cannot link the information well and thus sacrifice cognitive resources.

- Start by activating the students’ prior knowledge.
- Start with the perceptions and description of the patient and the situation and setting (see below). These observations can then be incorporated into the teaching and explanations as the students proceed.
- Avoid presentations or discussions of the entire patient case starting from the history, physical examination and diagnostics up to therapy, as this can be overwhelming, especially for inexperienced students.
- Focus on specific aspects of the patient’s case according to your learning objectives.
- Condense the material and organise it into meaningful parts so that the students can work within their limited processing capacity ( Mayer, 2010).

***Tip 4***: *Use visual thinking strategies*

Visual thinking strategies ( Yenawine, 2013) can be used to increase observational or visual diagnostic skills. An improvement in observational skills can serve as a vehicle to the development of crucial clinical competencies and encourage a more in-depth visual analysis that could be applied when observing a patient ( Cerqueira *et al.*, 2023; Hailey *et al.*, 2015). Allow a few moments of silent looking before beginning teaching and discussions.

- Allow an initial observation of the room, the patient and the setting: What do you see? What’s going on? (Ask once to initiate the discussion.)
- What do you see that makes you say that? (Ask whenever an interpretive comment is provided.)
- Ask repeatedly, ‘What more can we find?’ to extend the process and allow the group to find many possible answers.
- Facilitate the discussion ( Cerqueira *et al.*, 2023):
  - Listen carefully to catch everything that the students say while maintaining a neutral stance because this will leave the students free to find and think what they will. It also nurtures mutual respect among students, which is necessary for wide participation and risk-taking.
  - Point to observations as the students make comments.
  - Paraphrase each comment, and link related comments to surface commonalities and differences in interpretations.
  - Asking frequently throughout the discussion broadens and deepens the search for meaning, while linking allows the discussion to be coherent while honouring disparate ideas ( Hailey *et al.*, 2015).

***Tip 5:*** *Develop epistemological beliefs alongside the biopsychosocial model*

Epistemological beliefs at a personal level comprise a belief system about knowledge. It determines how (new) knowledge is perceived and processed ( Roex & Degryse, 2007). It is widely accepted that the biomedical model is still the dominant epistemology in medical education. It may thus be concluded that first-year students are more likely to view their knowledge of the natural sciences as more true and to rate the authority and expertise of science teachers as a source of truth compared to other disciplines ( Hofer, 2000). They have a more dualistically ‘right–wrong’ view of the world, in which faculty know the right answers ( Eastwood *et al.*, 2017). Students also tend to adopt the prevailing epistemology of their training environment ( Evans & Trotter, 2009). On the flip side, the biopsychosocial model attempts to integrate patients’ biological, psychological and social presentations into a coherent clinical whole.

- Take into account that especially students in the early semesters could perceive bedside teaching as fuzzy if a teacher does not give clear answers.
- Make epistemology explicit so that the students learn that there is not always a single ‘right’ answer to some problems and that multiple opinions may be equally valid ( Eastwood *et al.*, 2017).
- Work out the patient cases alongside the biopsychosocial model, which is accompanied by more uncertainty but can at least be associated with less stress reactions compared to a focused biomedical epistemology ( Evans *et al.*, 2012). This should take place against the background that uncertainties do not always have to result in an ongoing desire for clarification.

***Tip 6:*** *Set priorities in patient encounters*

Present the patient encounter in smaller explanatory and reflective segments. In this way, you can help the students construct scripts for later use as applicable patterns. Segmentation simplifies and saves on processing resources and improves comprehension ( Kurby & Zacks, 2008).

Medical teachers often feel obligated to discuss the entire patient case, starting from the history through to therapy. This can be but is not always useful. Students, particularly those at an early stage of their studies, need a greater degree of structure and scaffolding. Depending on the students’ levels of training, it could be more useful to select individual aspects of a patient that do not overload the students’ cognitive capacity and to go into more depth with regard to understanding and learning. Below are some examples for prioritisation.

- Focus on the patient’s illness perception, which is the cognitive representation or belief that a patient has about their illness. In fact, medical staff are usually unaware of patients’ ideas about their conditions, as staff rarely ask patients about their own ideas in clinical consultations ( Petrie *et al.*, 2007). Patients’ perceptions are often at variance with those of the medical staff. These perceptions have been found to be crucial determinants of behaviour and have been associated with a number of important outcomes, such as treatment adherence and functional recovery ( Broadbent *et al.*, 2015; Petrie *et al.*, 2007).
- Demonstrate and/or elaborate on a joint specified anamnesis for a patient complaint. What specific questions should I ask regarding the present complaint? How do I formulate them most reasonably? Reflect on the patient’s answers to your question.
- Elaborate on the physical findings (e.g. joint workup of a pleural effusion and its pathological and pathophysiological conditions). Put them in context with the patient’s complaints.
- Demonstrate/elaborate on questions that are target-oriented for differential diagnoses and further diagnostics procedures, which should be initiated.
- Incorporate short summaries in between. These can also be done by the students themselves: How did we get started? Let’s summarise what we have discussed so far. What has the patient said? What do you understand so far?

***Tip 7:*** *Demonstrate critical thinking and reasoning*

As an experienced physician, you often recognise patterns, match new findings and situations with these patterns and access stored application knowledge (scripts). You have learned to quickly grasp clinical findings and make intuitive decisions. Medical students cannot yet draw on such patterns – or at best, only to a limited extent. Note that pattern recognition can lead to incorrect conclusions and actions even among experts. Slow, analytical and critical thinking is always explicitly required when there are deviations from the pattern, and it supports hypothetical thinking ( Kahnemann, 2012).

Advanced students will have acquired increased knowledge and experience regarding the development of illness scripts, and they rely more on pattern recognition and heuristics for problem-solving. They are therefore more vulnerable to diagnostic errors resulting from availability bias and anchoring ( Royce *et al.*, 2019) than students in an earlier phase of their studies. These students lack sufficient experience to recognise and understand heuristic biases and may not immediately derive significant benefits from instruction in metacognitive techniques or debiasing strategies ( Royce *et al.*, 2019).

For advanced students, consider the following aspects in your teaching:

- Reflect and verbalise explicitly on cognitive biases in the decision-making process, such as
  - framing effects
  - adherence to first impressions or tentative diagnoses
  - failure to adjust diagnostic probabilities when presented with new data
  - judgement on the basis of recent case experiences
  - ignoring prior probabilities and base rates
  - the uncritical use of diagnostic test results.
- Demonstrate hypothetico–deductive reasoning by utilising the gathered information to test the hypotheses with the aim of either confirming or ruling out a hypothesis.
- Use questions to identify potential diagnostic possibilities and elaborate on specific distinguishing characteristics (semantic classifiers) to compare and contrast potential diagnoses for a given medical complaint (e.g.the chief complaint of ‘chest pain’ can be classified as acute or chronic, sharp or dull, constant or intermittent. It may or may not be associated with dyspnoea and can occur with or without multiple risk factors). These aspects are crucial for accurately representing the issue and determining their clinical significance to the historical elements in relation to the differential diagnosis ( Nierenberg, 2017).
- As you think about this, remember to involve the patient and explain it in a way that makes sense to them.
- Encourage students to understand these processes so that they can develop their critical thinking and clinical reasoning skills.
- Demonstrate Bayesian reasoning by using the base rate (pre-test) probabilities along with new clinical information (history, examination findings or test results) to calculate a revised (post-test) probability.

***Tip 8***: *Think aloud*

Thinking aloud is actually a research method used to study cognition. It may be helpful as a technique to enable students or inexperienced clinicians to learn from you by explicitly revealing your thought processes as an expert clinician. Students can there by observe how you utilise your knowledge to identify crucial information and establish connections and associations to organise them effectively ( Pinnock *et al.*, 2015).

- Remember to inform the patient in advance about your "think aloud" approach and its purpose, and then make sure to explain your thoughts to the patient in a way that they can understand afterward.
- Make your personal scripts transparent to address students’ frequently implicit queries related to specific history-taking and examination techniques. Unasked aspects of a medical presentation frequently hold the same importance as the ones that are enquired about ( Lubarsky *et al.*, 2015).
- Make students aware of how you determine the importance of specific features and label them as ‘particularly crucial’ when analysing a medical case ( Lubarsky *et al.*, 2015), ‘I palpate symmetrical lower leg oedema’, ‘I have to press slowly and for a prolonged period to move the fluid in the tissue away’, ‘Because the patient has been in bed for a longer time, the oedema may be less pretibial and more localised in the calves, dorsal femur and presacral’.

***Tip 9:*** *Use Socratic questioning*

Socratic questioning is a method that focuses on triggering answers by asking questions and is at the centre of critical thinking. Socratic questioning uses W-questions to help students generate answers for themselves. W-questions are questions about knowledge itself and are key to successful learning (e.g. What? When? Why?). This offers you as a teacher the opportunity to support the learning process by diagnosing the students’ misconceptions. You can give explanations if required, and you can correct those misconceptions in conversation.

- What was the point of your question?
- What are you thinking about? What were you thinking of?
- Why would you put an arterial line in the patient?
- Why are you thinking of a pulmonary embolism here?
- How can your assumptions bias your analysis and/or observations?
- Provide contrasts and ask students to compare different things (e.g. Why would you prescribe IV antibiotics and not oral antibiotics here?). Working with contrasts provides opportunities for teachers to diagnose students’ possible (mis)understandings.

***Tip 10:*** *Perform best-evidence clinical examinations*

No clinical test or measurement offers complete certainty regarding the existence or lack of a disease. It is crucial to consider the efficacy of clinical examination tests in distinguishing between different patients. The objective of clinical tests is not to achieve absolute diagnostic certainty but rather to diminish the degree of uncertainty. The integration of the most reliable evidence for each clinical test is vital for an accurate diagnosis. The scientific evaluation of clinical utility includes a comparison of the examination results with reference standards, such as X-ray examinations, and the use of statistical methods from the field of epidemiology ( Cleland *et al.*, 2020). Access literature that presents evidence of physical examination techniques (e.g. Cleland *et al.*, 2020; McGee, 2018).

- Be aware of the diagnostic properties of tests and measures.
- Consider the reliability and diagnostic utility of physical exam procedures.
- Consider likelihood ratios as the most useful diagnostic property to alter the probability that a patient has a specific disorder.

***Tip 11:*** *Conclude with a structured debriefing and feedback*

A debriefing is crucial for processing what has been learned and the possibility of a later transfer to another clinical situation. It usually takes place in close proximity to the patient in order to be able to supplement questions or follow-up examinations, if necessary. However, debriefings are often arbitrary and do not follow a particular structure. Structured debriefings are well established in simulated learning environments and are used in real clinical contexts as well ( Bajaj *et al.*, 2018; Dreifuerst, 2015; Phillips *et al.*, 2023). The perspective is backwards and about decompression (emotional processing), analysis (clarifying questions), evaluation and feedback. Several issues should be considered.

- Create a positive atmosphere.
- Collect the students’ reactions. If necessary and useful, the thematisation of emotions should take priority so as not to overshadow the factual discussion. This depends on the experience of the patient encounter (e.g. the severity of the illness, the behaviour of the patient, the severity or speciality of the observed findings).
- Trigger a recap by the students. Let them describe the patient’s presentation again: Can you briefly summarise the case? What were the working hypotheses? How did the patient react when I asked him? What findings did we collect?
- Analyse the case with the students: How can our examination findings be explained? How do they fit together? How do the findings fit the patient's history?
- Generalise and transform the content from the specific case by addressing guidelines, for example: If you were to meet a patient tomorrow with similar complaints, how would you proceed?

## Conclusion

The eleven tips may not always be fully implemented in each bedside teaching, but they should be selectively applied for targeted students. They are more to be understood as a repertoire of methods. Bedside teaching takes place in the presence of a patient and in a clinical setting. This can only be planned to a certain extent, and its realisation may deviate from the planning. Bedside teaching is a highly social interaction, and the sociology literature questions the assumption that people are rational decision-makers. Instead of weighing the pros and cons of a decision objectively and logically, the model of social processes emphasises the effects of the broader context on how decisions are made ( Bruch & Feinberg, 2017). The presented tips are therefore intended to reveal the processes of clinical reasoning and decision-making for patients in a more rational, structured, analytical and critical manner by taking learning theories into account.

## Data Availability

No data are associated with this article.
