# Practical tips to improve bedside teaching using learning theories and clinical reasoning

**DOI:** 10.12688/mep.19826.2

**Published:** 2024-03-11

**Authors:** Thomas Rotthoff

**Affiliations:** 1 Medical Didactics and Education Research (DEMEDA), Augsburg University, Augsburg, 86259, Germany

**Keywords:** bedside teaching, practical tips, medical education bedside teaching

## Abstract

Bedside teaching strengthens the link between theory and practice. The tips given here, which were derived from various learning theories and models, aim to provide structure to bedside teaching and to make this format effective, even though empirical evidence is still missing for this specific setting. These 10 tips may not always be fully implemented in each bedside teaching, but they should be applied selectively for targeted students. In essence, they are more to be understood as a repertoire of effective methods and are intended to expand the literature and framework concepts already available.

## Introduction

Unlike other teaching formats, bedside teaching is authentic and the only format in medical education in which the skills of history-taking and physician–patient communication, physical examination, clinical reasoning, decision-making, empathy and professionalism can be simultaneously taught and learned as an integrated process in a real clinical setting ( Garout *et al.*, 2016; Spencer, 2003; Verghese *et al.*, 2011). Particularly in competency-based medical education, bedside teaching is suitable for promoting, monitoring and assessing students’ continuous development in competence and performance. Notably, it can take the form of an individual or collective learning via practice-based experiences ( Yardley *et al.*, 2012). Bedside teaching provides students with the opportunity to understand the patients’ personal experiences and perceptions of the disease, including the symptoms. Additionally, it enables patients to gain insights into their conditions and treatments. Theories suggest the value of involving patients at an early stage of a student’s medical education ( Dornan *et al.*, 2006; Wenrich *et al.*, 2013; Yardley *et al.*, 2013) because early experiences with patients can help students become familiar with the medical profession and strengthen their learning and skills acquisition ( Dornan *et al.*, 2006). Accordingly, bedside teaching should not be delivered uniformly to all students; instead, it should be student-centred ( Ende, 1997) and adapted to the students’ levels of experience. Students in the initial phases of their studies require more structured guidance and support from educators. This assistance, which is generally referred to as scaffolding, is crucial for tasks that may be beyond a student’s existing capabilities ( van de Pol *et al.*, 2010). As noted by Nevalainen *et al.* (2010) and Evans *et al.* (2012), the need for scaffolding arises from several factors, including the students’ levels of knowledge, uncertainties about their professional competencies, the realisation of the imprecision in medicine and an increased sense of responsibility towards patient care. Bedside teaching is facing growing challenges today ( Peters & Ten Cate, 2014; Qureshi & Maxell, 2012) due in part to structural changes in medical care. It is also often undervalued and recognised as a demanding instructional method in medical education that transcends the role of merely practising medicine in the presence of students. The current paper seeks to augment Yardley *et al.*’s (2012) comprehensive insights on experiential learning within theoretical frameworks and offers practical advice for the realisation of bedside teaching based on learning theories. Thereby it is essential to take into account that learning is context-specific and closely related to the learning environment, the content and the instructional strategies employed ( Yardley *et al.*, 2012).

### Tip 1

***Consider the influence of emotion on learning***

According to attentional control theory ( Eysenck *et al.*, 2007), anxiety and stress impair attention-shifting task performance. Anxious individuals exert considerable effort to sustain efficiency, which taxes the limited capacity of their working memory’ ( Edwards *et al.*, 2015). Evidence also indicates that a state of anxiety reduces working memory capacity ( Moran, 2016; Ward *et al.*, 2020). As more resources are used to achieve a certain level of performance, efficiency declines. For students in the first semesters of their studies, the clinical environment and routine are often not familiar, and patients’ conditions and medical histories may trigger emotions that have not yet been processed professionally. The uncertainties associated with standing in a white coat in a ward and the expectations of the clinical teacher can also contribute to students’ anxiety. In a performance situation, a person needs to integrate their cognitive and affective resources and skills dynamically. Excitement and emotion can influence learning by affecting students’ attention, motivation and self-regulation. Take into account that memories of negative experiences are generally better remembered than positive ones and experiences that evoke strong emotions are also remembered more easily than those that are less stimulating. If bedside teaching triggers emotions, students remember details of this event more accurately than experiences that do not have an emotional component ( McConnell & Eva, 2023). The same can also be postulated for the patient's experiences in bedside teaching. This makes it all the more important to treat patients as equal members of the group and to give them an active role during bedside teaching. However, bedside teaching also provides a unique opportunity to foster learning by triggering emotions, e.g. by arousing enthusiasm and curiosity for the respective subject area or feelings of belonging and cooperation in the exchange of experiences.

- Build a relationship with your students, and create a safe learning environment with an anxiety-free atmosphere. Encounter the students and patients as equals.
- Start with the students’ existing competence and skills levels to help them regulate their emotions. Activate the students’ prior knowledge.
- Support the students by naming and interpreting stressors so that they result in eustress rather than distress ( Jamieson *et al.*, 2013; Rudland *et al.*, 2020).
- Reduce extraneous stressors, such as personal pressure to do well (e.g. ‘You can’t make any mistakes; we will work this out together with the patient right away’).
- Promote stressors that are more likely to facilitate learning and cognitive performance by, for example, reducing the complexity of the situation.
- Keep in mind that students have different mindsets, personality traits and self-beliefs regarding how to react in challenging situations.

### Tip 2

***Start with a briefing***

Surprisingly, scant evidence exists regarding the impact of briefings in real workplace learning environments, although some studies have demonstrated their effectiveness in simulated contexts ( Tyerman *et al.*, 2019). Although simulation training differs from real workplace experience, the principles of some learning theories appear applicable in actual clinical situations, particularly when compared to complex simulation scenarios. Schema theory, which was introduced by Bartlett in 1932, suggests that new information requires memory adaptation, with schemata facilitating the categorisation of this information. Schemata shape how we focus on and understand new information ( Bartlett, 1995) and involves a reduction process, where complex information is assimilated or integrated into higher order structures, often unconsciously during everyday perception ( Wirtz *et al.*, 2017). From a more functional perspective, learning is viewed as an ever-evolving process of becoming, without schemas ever being frozen in a long-term state of being ( Iran-Nejad & Winsler, 2000). In line with this theory, an effective briefing could help facilitate the integration of information into existing knowledge and experiential schemata by activating prior knowledge before new information is presented.

Briefings can boost competence and relatedness by establishing a good learning atmosphere and by outlining clear objectives and learning goals to seem attainable, which thus serves students’ needs. These aspects can be derived from Self-determination theory ( Deci & Ryan, 1985), which posits that autonomy, competence and relatedness enhance motivation. A briefing takes place in close proximity to the patient, typically in front of the patient’s room.

- Link information to your students’ prior knowledge, as this can make it easier for them to integrate new data into existing schemata. The recognition of prior knowledge involves a reflective process, where students connect their previous learning to the patient encounter and thereby reorganise, solidify, enhance and situate their knowledge within emerging skills ( Dornan *et al.*, 2019).
- Address the learning objectives.
- Reduce content by providing your students with only the essential information about the patient and the setting: What can the students expect to see and experience behind the door? What kind of patient will they see, and what is the patient’s mental and physical condition? Furthermore,
- What do you expect from your students? Do they have to perform a task or demonstrate something on the patient? In what manner? What will the students’ specific roles be?
- Give advice on special instructions (e.g. hygiene or even issues to be avoided).
- Move more detailed information about the patient or pathophysiological explanations to the encounter, debriefing or detached teaching sessions.

### Tip 3

***Reduce the cognitive load***

Cognitive load theory ( Sweller, 2011) highlights the capacity limit of working memory, which means that the volume of information that can be processed at once is limited. Working memory is responsible for actively processing and handling information in real time and receives inputs from both sensory systems (vision, touch, hearing) and long-term memory. The process of ‘chunking’, which means grouping information into recognisable patterns, enhances memory storage capacity ( Cowan, 2014; Miller, 1956). As an expert, your ‘chunks’ are already considerably well developed, but students can be overwhelmed in situations that you as a clinician process automatically due to your clinical expertise. Supposedly simple cases can be complex for students and overload can occur when students are confronted with environments and dynamic visual and emotional situations with which they are not familiar (e.g. patients with many catheters or monitoring systems, abnormal physical findings) and distractions, such as other patients or family members in the room. The greater the prior knowledge and familiarity with an environment, the better working memory can draw on information stored in a person’s long-term memory, which is already organised. If students have only little prior knowledge or experience, they cannot link the information well and thus sacrifice cognitive resources.

- Give your students time to orient themselves in the setting.
- Avoid presentations or discussions of the entire patient case starting from the history, physical examination and diagnostics up to therapy, as this can be overwhelming, especially for inexperienced students.
- Focus on specific aspects of the patient’s case according to your learning objectives.
- As for briefings, activate your students’ prior knowledge during the encounter with the patient.
- Condense the material and organise it into meaningful parts so that the students can work within their limited processing capacities ( Mayer, 2010).

### Tip 4

***Use visual thinking strategies***

Visual thinking strategies ( Yenawine, 2013) were initially used to refer to the development of visual literacy, which was attained by detailed observation of representational paintings, but they have found their way into medical programs in recent years. In effect, the experiential process of seeing visual details in paintings can be applied among medical students to develop their diagnostic skills ( Dolev *et al.*, 2001; Naghshineh *et al.*, 2008; Schaff *et al.*, 2011). When practising the method, the following questions are typically asked: What is going on in the picture? What do you see that makes you say that? What more can we find? These questions were developed based on empirical research ( Yenawine, 2013) to keep groups engaged in the process and to encourage participants to look for visual evidence to support their expressed conclusions. This process makes students take more time to look carefully and to back up their first thoughts with proof based on what they see. In doing so, students learn to generate better and more thorough solutions to problems ( Schaff *et al.*, 2011). Although hardly any studies have specifically explored the use of the aforementioned questions in bedside teaching, the rationale supporting these questions suggests that applying them could enhance students’ observational and visual diagnostic skills. An improvement in observational skills can serve as a vehicle to the development of crucial clinical competencies and encourage a more in-depth visual analysis, which could be applied when observing a patient ( Cerqueira *et al.*, 2023; Hailey *et al.*, 2015).

- Allow an initial observation of the room, the patient and the setting
  - Explain your students’ observations to the patient, and categorise them in a way the patient can understand. As the process requires them to look longer and to support their initial ideas with evidence from their observations, students learn the value of postponing a seemingly inevitable conclusion in order to deepen and broaden the solutions to the problem at hand.
  - Explain the procedure to the patient and the students, as this differs from a classic visit or medical examination.
  - What’s going on? (Ask once to initiate the discussion.) These observations can then be incorporated into the teaching and explanations as the students proceed.
  - What do you see that makes you say that? (Ask whenever an interpretive comment is provided.)
- Ask repeatedly, ‘What more can we find?’ to extend the process and allow the group to find many possible answers.
- Facilitate the discussion ( Cerqueira *et al.*, 2023):
  - Listen carefully to catch everything that the students say while maintaining a neutral stance because this will leave them free to find and think what they will. It also nurtures mutual respect among the students, which is necessary for wide participation and risk-taking.
  - Point to observations as the students make comments.
  - Paraphrase each comment, and link related comments to surface commonalities and differences in interpretations. Linking allows the discussion to be coherent while honouring disparate ideas ( Hailey *et al.*, 2015).
  - Ask questions frequently throughout the discussion. This broadens and deepens the search for meaning.

### Tip 5

***Develop epistemological beliefs alongside the biopsychosocial model***

Epistemological beliefs at a personal level comprise a belief system about knowledge. They determine how (new) knowledge is perceived and processed ( Roex & Degryse, 2007).

The biomedical model remains the prevailing epistemological framework in medical education, and its use influences students at the beginning of their studies to prioritise natural sciences as the most valid knowledge. This can lead to a binary ‘right–wrong’ worldview, where students believe faculty know and possess the definitive and right answers ( Eastwood *et al.*, 2017; Hofer, 2000). Students also tend to adopt the prevailing epistemology of their training environment ( Evans & Trotter, 2009). On the flip side, the biopsychosocial model attempts to integrate patients’ biological, psychological and social presentations into a coherent clinical whole. Integrating a more nuanced understanding of medical issues involves embracing the complexity of human health, as seen in the biopsychosocial model ( Evans *et al.*, 2012). Take into account that students, especially in the early semesters, could perceive bedside teaching as somewhat fuzzy if uncertainties arise from the case and not all questions can be answered clearly. This is because they will predominantly be accustomed to the basic sciences.

- Work out the patient case alongside the biopsychosocial model. This may generate more uncertainty but is at least associated with fewer stress reactions compared to a focused biomedical epistemology ( Evans *et al.*, 2012). For example, students often focus on the physical causes of pain but addressing psychological and social factors and patient preferences can offer a more holistic treatment approach that acknowledges the potential for multiple solutions.
- Clarify epistemology to teach your students that some problems lack a single ‘right’ answer, and multiple perspectives can be valid ( Eastwood *et al.*, 2017). For instance, this can be achieved by explaining your methods for handling uncertainty, integrating evidence with your clinical judgment and viewing mistakes as vital learning opportunities by applying open discussions and analysing any errors that may also have occurred in the present case.

### Tip 6

***Set priorities in patient encounters***

The sociocultural theory of human learning describes learning as a social process in which the potential for cognitive development is limited to social interactions and a ‘zone of proximal development’. In the latter, which is also described as ‘supported participation’, the student is prepared for new knowledge and experiences but needs support to develop fully ( Billett, 2002). This means that educators provide structured support to students as they acquire complex clinical skills and knowledge, with the assistance gradually being reduced as the students’ competence increases. Accordingly, it could be helpful to present the patient encounter in smaller explanatory and reflective segments. Segmentation simplifies and saves on processing resources and improves comprehension ( Kurby & Zacks, 2008). Medical teachers often feel obligated to discuss the entire patient case, starting from the history through to therapy. This can be but is not always useful. Depending on the students’ levels of training, it may be more useful to select individual aspects of a patient that do not overload the students’ cognitive capacity and to go into more depth to enhance their understanding and learning. Below are some examples for selection and prioritisation.

- Focus on the patient’s illness perception, which is the cognitive representation or belief that a patient has about their illness. In fact, medical staff are usually unaware of patients’ ideas about their conditions, as staff rarely ask patients about their own ideas during clinical consultations ( Petrie *et al.*, 2007). Patients’ perceptions are often at variance with those of medical staff. However, these perceptions have been found to be crucial determinants of behaviour and have been associated with a number of important outcomes, such as treatment adherence and functional recovery ( Broadbent *et al.*, 2015; Petrie *et al.*, 2007).
- Demonstrate and/or elaborate on a joint specified anamnesis for a patient complaint. For example, what specific questions should I ask regarding the present complaint of dysnpea? How do I formulate them most reasonably? Reflect on the patient’s answers to your questions.
- Elaborate on the physical findings (e.g. joint workup of a pleural effusion and its pathological and pathophysiological conditions). Put them in context with the patient’s complaints.
- Demonstrate/elaborate on questions that are target-oriented for differential diagnoses and further diagnostics procedures, which should be initiated.
- Incorporate short summaries in between. These can also be done by the students themselves: How did we get started? Let’s summarise what we have discussed so far. What did the patient say? What do you understand so far? Also report the clinical progress to the patient in an understandable way.

### Tip 7

***Demonstrate critical thinking and clinical reasoning***

As an experienced physician, you often recognise patterns, match new findings and situations with these patterns and access stored application knowledge (scripts). You will have learned to quickly grasp clinical findings and make intuitive decisions. Medical students cannot yet draw on such patterns – or at best, can only do so to a limited extent. Note that pattern recognition can lead to incorrect conclusions and actions even among experts. Slow, analytical and critical thinking is always explicitly required when there are deviations from the pattern, and it supports hypothetical thinking ( Kahnemann, 2012).

Advanced students will have acquired increased knowledge and experience regarding the development of illness scripts, and they rely more on pattern recognition and heuristics for problem-solving. They are therefore more vulnerable to diagnostic errors resulting from availability bias and anchoring ( Royce *et al.*, 2019) than students in an earlier phase of their studies. These advanced students lack sufficient experience to recognise and understand heuristic biases and may not immediately derive significant benefits from instruction in metacognitive techniques or debiasing strategies ( Royce *et al.*, 2019). For advanced students, consider incorporating the following aspects in your teaching:

- Reflect and verbalise explicitly on cognitive biases in the decision-making process, such as
  - framing effects
  - adherence to first impressions or tentative diagnoses
  - failure to adjust diagnostic probabilities when presented with new data
  - judgement on the basis of recent case experiences
  - ignoring prior probabilities and base rates
  - the uncritical use of diagnostic test results
- Demonstrate hypothetico–deductive reasoning by utilising the gathered information to test the hypotheses with the aim of either confirming or ruling out a hypothesis.
- Use questions to identify diagnostic possibilities and elaborate on specific distinguishing characteristics (semantic classifiers) to compare and contrast potential diagnoses for a given medical complaint (e.g. The chief complaint of ‘chest pain’ can be classified as acute or chronic, sharp or dull, constant or intermittent. It may or may not be associated with dyspnoea and can occur with or without multiple risk factors.) These aspects are crucial for accurately representing the issue and determining their clinical significance to the historical elements in relation to the differential diagnosis ( Nierenberg, 2017).
- Encourage the students to understand these processes so that they can develop their critical thinking and clinical reasoning skills. Provide feedback not only on the students’ clinical skills but also their reasoning processes. Highlight effective reasoning strategies, and identify areas for improvement in how they construct and apply their knowledge ( Schön, 1991).
- Demonstrate Bayesian reasoning by using the base rate (pretest) probabilities along with new clinical information (history, examination findings or test results) to calculate a revised (posttest) probability.

### Tip 8

***Think aloud***

Think aloud is derived from information processing theory and discloses cognitive processing and development ( Banning, 2008). During thinking aloud, working memory thoughts are transformed into spoken words ( Ericsson & Simon, 1980; Johnson *et al.*, 2023), which means that a person does *not* describe or explain what they are thinking but simply verbalises how they are using the available information to generate a solution to a problem ( Pinnock *et al.*, 2015). Despite limited published research on think aloud in bedside teaching, as noted by Siddiqui (2014), its effectiveness in assessing postgraduate trainees’ clinical reasoning indicates its potential usefulness in this context. This approach requires some training.

- Enable your students or inexperienced clinicians to learn from you by explicitly revealing your thought processes as an expert clinician.
- The students can thereby observe how you utilise your knowledge to identify crucial information and establish connections and associations to organise them effectively ( Pinnock *et al.*, 2015).
- Make your personal scripts transparent to address your students’ frequently implicit queries related to specific history-taking and examination techniques. Unasked aspects of a medical presentation frequently hold the same importance as the ones that are enquired about ( Lubarsky *et al.*, 2015)
- Make your students aware of how you determine the importance of specific features and label them as ‘particularly crucial’ when analysing a medical case ( Lubarsky *et al.*, 2015); for example: ‘I palpate symmetrical lower leg oedema’, ‘I have to press slowly and for a prolonged period to move the fluid in the tissue away’, ‘Because the patient has been in bed for a longer time, the oedema may be less pretibial and more localised in the calves, dorsal femur and presacral’.

### Tip 9

***Use Socratic questioning***

Socratic questioning stimulates answers through inquiry. It is based on constructivism theory, which posits that knowledge is not merely transmitted but is actively constructed by integrating new information into existing cognitive frameworks, so that unique, significant subject-specific knowledge is gained ( Tenorth & Tippelt, 2012). Socratic questioning uses W-questions to help students generate answers for themselves. W-questions query knowledge itself (e.g. What? When? Why?) and require students’ active engagement to facilitate and construct a more profound comprehension of the subject matter. This offers you as a teacher the opportunity to support the learning process by diagnosing your students’ misconceptions. You can give explanations, if required, and you can correct their misconceptions in conversation. For successful engagement with these questions, it is crucial to establish a trusting learning environment where your students feel valued and respected and are seen as equals, as this encourages their willingness to participate. It is also helpful to clarify the purpose of these questions beforehand, namely, that they aim to reveal individual thought processes, which will allow all the other students to follow along and to promote a joint learning process.

- What are you thinking about? What were you thinking of?
- What was the point of your question?
- Why would you put an arterial line in the patient?
- Why are you thinking of a pulmonary embolism here?
- How can your assumptions bias your analysis and/or observations?
- Provide contrasts, and ask the students to compare different things (e.g. Why would you prescribe intravenous antibiotics and not oral antibiotics here?). Working with contrasts provides opportunities for teachers to diagnose students’ possible (mis)understandings.

Asking questions can also stimulate reflection-in-action during bedside teaching. Schön’s (1991) reflective learning theory posits that real-time reflection helps identify and solve problems as they occur. Encouraging your students to learn from their experiences and reactions can enhance their skills and effectiveness. Examples of questions to trigger reflection-in-action are as follows:

- What do you perceive right now during your examination, and how do you interpret your perceptions?
- When you just realised that the patient is unable to sit up for the physical examination, how did you adapt your actions?
- What alternative strategies could you use in this situation and why?

### Tip 10

***Conclude with a structured debriefing and feedback***

Despite the lack of a common definition ( Winchester-Seeto & Rowe, 2019), debriefing is recognised as an essential analytical process that enhances learning by having students reflect on their experiences ( Lederman, 1992). Debriefings usually take place in close proximity to the patient to supplement questions or conduct follow-up examinations, if necessary. Debriefing is well acknowledged in both simulation training ( Dreifuerst, 2015) and bedside teaching ( Carlos *et al.*, 2016; Phillips *et al.*, 2024; Ramani, 2003), but unlike for simulation environments, there is a significant shortage of scientific evidence regarding its effects in the context of bedside teaching. However, it can be assumed that the effectiveness of debriefing in bedside teaching can be derived from various theoretical concepts, such as experiential learning by Kolb *et al.* (2000), reflective learning by Schön (1991) and the theories of social learning by Bandura and Walter (1977).

Debriefing follows the concept of reflective thinking on experiential learning and facilitates guided reflection to bridge the gap between experience and its meaningful understanding to reinforce learning ( Fanning & Gaba, 2007). It provides students with the opportunity to consciously review their actions, thereby allowing them to verbalise their thoughts on the consequences of their actions or lack thereof ( Dufrene & Young, 2014; Raemer *et al.*, 2011). This approach may enhance their ability to apply these actions in various clinical scenarios, including future transfers to different clinical settings.

- Prompt your students to provide a recap of the patient’s presentation and the concrete experience.
  - What happened?
  - What were your initial thoughts and feelings? Prioritise discussing emotions if it will enhance understanding, especially those based on the context of the patient encounter, such as the illness severity, the patient’s behaviour and unique findings.
  - What findings did you collect? How can your examination findings be explained?
  - How do they fit together? How do the findings fit the patient’s history?
  - Can you briefly summarise the case?
  - What were the working hypotheses?
- Trigger reflective observation.
  - Which parts grabbed your attention or surprised you, and why do you think that is?
  - How did the patient react when we explained our findings?
  - How did the patient react when I asked (…)?
- Stimulate your students to develop an abstract conceptualisation.
  - How can you apply what you have learned in similar situations?
  - Generalise and transform the content from the specific case by addressing guidelines; for example: if you were to meet a patient with similar complaints tomorrow, how would you proceed?

In Addition Schön’s model (1991) also notes that reflection-on-action is essential for professional growth where questions such as these can be derived helpfully:

- What can you conclude from the experience you have just had? What did you learn?
- What would you keep the same or change next time?

By observing you as an experienced practitioner, your students can improve their clinical skills and professional behaviour, which enables personalised learning and their future behaviours. Bandura and Walters (1977) emphasised the significance of social learning through observation, modelling and imitation.

- What did you notice about my/our communication with the patient? How did I try to address the patient’s concerns?
- How and where did I position myself during the encounter?
- How did I talk to the patient during the physical examination?
- Did you notice any particularly challenging moments when interacting with the patient?
- Are there any bedside teaching moments you’d like to adopt in your own practice?
- What actions or methods observed during the session do you think you could use in your own practice, and how would you apply them?

## Conclusion

Bedside teaching plays a central role in medical education by strengthening the link between theory and practice and fostering the integration of the skills that are essential for future healthcare professionals. The tips given here, which were derived from various learning theories and models, aim to provide structure to bedside teaching and to make this format effective, even though empirical evidence is still missing for this specific setting. The tips should demonstrate the importance of accommodating the diverse experience and learning needs of your students, stimulate reflective learning and emphasise the importance of creating a supportive and student-centred environment that supports the acquisition of clinical skills as well as critical thinking and professionalism. These 10 tips may not always be fully implemented in each bedside teaching, but they should be applied selectively for targeted students. In essence, they are more to be understood as a repertoire of effective methods.

## Data Availability

No data are associated with this article.
