# Supplementary material for: Practical tips to improve bedside teaching using learning theories and clinical reasoning
Source: MedEdPublish (2016). 2024 Mar 11;13:215. Originally published 2023 Oct 12. [Version 2] doi: 10.12688/mep.19826.2 (PMC11069040; doi:10.12688/mep.19826.2)
Supplement: Peer review history

# Reviewer report: Reviewer response for version 2

**DOI:** 10.21956/mep.21702.r36221

**Published:** 2024-05-03

**Authors:** Marjel van Dam

**Affiliations:** 1 University Medical Center Utrecht, Utrecht, The Netherlands

I extend my congratulations to Thomas Rotthoff for this improved version. He has effectively responded to the comments raised on the previous iteration. With this new approach, the scientific field of bedside teaching has definitely broadened.

If evidence from practice is presented, are all the underlying source data available to ensure full reproducibility?

Partly

Is the topic of the practical tips discussed accurately in the context of the current literature

Partly

Are all factual statements correct and adequately supported by citations?

Partly

Are the conclusions drawn balanced and justified on the basis of the presented arguments?

Partly

Are arguments sufficiently supported by evidence from the published literature and/or the authors’ practice?

Partly

Reviewer Expertise:

Medical education: entrustable professional activities, bedside teaching, clinical supervision

I confirm that I have read this submission and believe that I have an appropriate level of expertise to confirm that it is of an acceptable scientific standard.

# Reviewer report: Reviewer response for version 2

**DOI:** 10.21956/mep.21702.r36222

**Published:** 2024-04-05

**Authors:** Subha Ramani

**Affiliations:** 1 Brigham and Women's Hospital, Harvard Medical School, Boston, USA

The author has satisfactorily responded to my comments and suggestions. I am happy to add my approval

If evidence from practice is presented, are all the underlying source data available to ensure full reproducibility?

Not applicable

Is the topic of the practical tips discussed accurately in the context of the current literature

Yes

Are all factual statements correct and adequately supported by citations?

Yes

Are the conclusions drawn balanced and justified on the basis of the presented arguments?

Yes

Are arguments sufficiently supported by evidence from the published literature and/or the authors’ practice?

Yes

Reviewer Expertise:

Clinical teaching, feedback, inclusion in education, mentorship

I confirm that I have read this submission and believe that I have an appropriate level of expertise to confirm that it is of an acceptable scientific standard.

# Reviewer report: Reviewer response for version 1

**DOI:** 10.21956/mep.21242.r35045

**Published:** 2023-11-13

**Authors:** Marjel van Dam

**Affiliations:** 1 University Medical Center Utrecht, Utrecht, The Netherlands

As a clinician and medical educator, I have a keen interest in optimizing my teaching methods, particularly when it comes to bedside teaching. This is why I readily agreed to review the article "Practical Tips to Enhance Bedside Teaching Using Learning Theories and Critical Reasoning" by Thomas Rotthoff. In this work, Rotthoff emphasizes the significance of delving into the cognitive theories that underlie bedside teaching, which is a commendable approach.

 Rotthoff isn't the first author to offer tips on bedside teaching in recent decades. He has authored this article to provide practical guidance for revealing the processes of clinical reasoning and decision-making in a more rational, structured, analytical, and critical manner. It's clear that his focus differs from previous articles, such as those by Ramani in "Twelve Tips to Improve Bedside Teaching" (2003), Ray's "Bedside Teaching" (2009), Carlos's "Teaching at the Bedside: Maximal Impact in Minimal Time" (2016), and Santhosh's "Practical Tips for ICU Bedside Teaching" (2018).

 Bedside teaching has the potential to be a win-win-win situation. One of the benefits is that it provides an opportunity for everyone involved—students, teachers, and patients—to gain valuable insights. (See "Bedside Teaching Pearls" by Ramani S, Heublein M, Kryzhanovskaya E, The Curbsiders Teach Podcast, February 22, 2022.)

 However, Rotthoff seems to have a narrower focus. In his introduction, he makes several bold statements that lack sufficient supporting references. He refers to a personal publication that suggests bedside teaching is under pressure today, but this isn't the most reliable source to substantiate this claim. He also asserts that students at an early stage of their studies require more structure and scaffolding, without citing supporting literature.

 Rotthoff primarily focuses on medical students, which, in today's healthcare setting, might be seen as somewhat limiting. It would be intriguing to explore how learning theories could benefit patients, but Rotthoff doesn't address this.

 I'd like to delve into some of the tips that, in my view, require further clarification or elaboration.

- Tip 1: Consider the Role of Emotions in Learning. Rotthoff repeatedly refers to a review by Tyng, which is a general psychological review not specifically focused on early-stage medical students. He doesn't touch on, for instance, McConnell's work (AM 2016) Impact of Emotions on Learners," nor does he address the emotions experienced by patients in clinical encounters, an aspect often overlooked by medical professionals. Teacher emotions also go unmentioned.
- Tip 2: Begin with a Debriefing. This is just one part of the initial steps in bedside teaching. Both Ramani and Carlos place debriefing in a broader context that includes the patient and emphasizes creating a safe learning environment. Rotthoff's references are primarily related to simulation settings, which, while important, might not align with the primary focus of bedside teaching, in my opinion. However, a safe learning environment remains essential in every setting.
- Tip 4: Utilize Visual Thinking Strategies. This tip finds support in a systematic review by Cerqueira, which cautiously concludes that the VTS approach can foster essential clinical competencies and encourage more in-depth visual analysis when observing a patient. Hailey et al.'s chapter discusses two studies that demonstrate an impact on healthcare education. Both do not mention bedside teaching.
- Tip 5: Develop Epistemological Beliefs Alongside the Biopsychosocial Model. This tip requires further clarification, especially in the context of bedside teaching. Most clinicians may not fully grasp this theory, let alone be able to address it in a meaningful way. Rotthoff doesn't mention continuous professional development for clinical teachers, and, in my humble opinion, his expectations of the average clinician involved in bedside teaching might be a little too high.
- Tip 6: Set Priorities in the Patient Encounter. This is part of the preparation process advocated by Ramani and Carlos, but it's more comprehensive.
- Tip 8: Think Aloud. The current support from the literature for thinking aloud in bedside teaching seems to lack weight.
- Tip 9: Use Socratic Questioning. Similar to tip 5, this also requires training for clinicians. If not executed correctly, Socratic questioning may be perceived as "pimping," embarrassing learners and reinforcing the teacher's position of power over the team, potentially having the opposite effect.
- Tip 10: Perform Evidence-Based Clinical Examination. In my opinion, this tip stands out when discussing cognitive theories as the foundation for bedside teaching.
- Tip 11: Conclude with a Debriefing and Feedback. This insight isn't novel and has been mentioned by numerous authors, including Ramani and Carlos, who place it in a broader context that includes the patient.

The conclusion section is formulated cautiously and serves more as a summary than a true conclusion. A conclusion should build upon the information presented in the rest of the article, and including a reference in it does not strengthen the conclusion.

 In summary, I genuinely appreciate Rotthoff's novel approach to improving bedside teaching. However, the current draft of this personal perspective on how to enhance bedside teaching lacks the substantiation and scientific rigor necessary to implement the suggested tips and truly enhance bedside teaching.

If evidence from practice is presented, are all the underlying source data available to ensure full reproducibility?

Partly

Is the topic of the practical tips discussed accurately in the context of the current literature

Partly

Are all factual statements correct and adequately supported by citations?

Partly

Are the conclusions drawn balanced and justified on the basis of the presented arguments?

Partly

Are arguments sufficiently supported by evidence from the published literature and/or the authors’ practice?

Partly

Reviewer Expertise:

Medical education: entrustable professional activities, bedside teaching, clinical supervision

I confirm that I have read this submission and believe that I have an appropriate level of expertise to state that I do not consider it to be of an acceptable scientific standard, for reasons outlined above.

# Response

**Published:** 2024-03-01

**Authors:** Thomas Rotthoff

**Affiliations:** Medical Didactics and Education Research (DEMEDA), Augsburg University, Augsburg, Bavaria, Germany

Many thanks  for your critical and constructive review. I am pleased about the generally positive feedback on deriving tips for bedside teaching from learning theory.Your comments stimulated me to revise the article comprehensively. I would like to specify, that rather than presenting a new overarching *poposal for the implementation of bedside taching in general, the work at hand just aims to broaden the perspective on bedside teaching. It aims to supplement the currently available literature with practical tips that are derived from learning theory. This is intended to enable a better understanding of the underlying theoretical principles and to contribute to its implementation.* *It should make practical implementation more comprehensible. * As a clinician and medical educator, I have a keen interest in optimizing my teaching methods, particularly when it comes to bedside teaching. This is why I readily agreed to review the article "Practical Tips to Enhance Bedside Teaching Using Learning Theories and Critical Reasoning" by Thomas Rotthoff. In this work, Rotthoff emphasizes the significance of delving into the cognitive theories that underlie bedside teaching, which is a commendable approach.

 Rotthoff isn't the first author to offer tips on bedside teaching in recent decades. He has authored this article to provide practical guidance for revealing the processes of clinical reasoning and decision-making in a more rational, structured, analytical, and critical manner. It's clear that his focus differs from previous articles, such as those by Ramani in "Twelve Tips to Improve Bedside Teaching" (2003), Ray's "Bedside Teaching" (2009), Carlos's "Teaching at the Bedside: Maximal Impact in Minimal Time" (2016), and Santhosh's "Practical Tips for ICU Bedside Teaching" (2018). The aim of this work is not to introduce a “new” overarching model on bedside teaching that it is not either, but rather to extend and enrich the existing body of literature. 

 Bedside teaching has the potential to be a win-win-win situation. One of the benefits is that it provides an opportunity for everyone involved—students, teachers, and patients—to gain valuable insights. (See "Bedside Teaching Pearls" by Ramani S, Heublein M, Kryzhanovskaya E, The Curbsiders Teach Podcast, February 22, 2022.)   I fully agree that bedside teaching can be valuable not only for students, but also for teachers and patients. I have now put more emphasis on this, yet I would like to keep the focus on the students since the present work should about concrete didactic and practical tips for teacher derived from learning theory. I hope the text now makes this clearer. 

 However, Rotthoff seems to have a narrower focus. In his introduction, he makes several bold statements that lack sufficient supporting references. He refers to a personal publication that suggests bedside teaching is under pressure today, but this isn't the most reliable source to substantiate this claim.   Thank you, I appreciate the criticism. I have now referred to specific works in the introduction: Qureshi and Maxell 2012 and a review by Peters and Ten Cate 2014.   He also asserts that students at an early stage of their studies require more structure and scaffolding, without citing supporting literature.   I added references to substantiate the statement (see introduction: Van de Pol et al. 2010; Braun et al. 2017). Accordingly, I have integrated this aspect in the tips as they correspond to a guided learning. 

 Rotthoff primarily focuses on medical students, which, in today's healthcare setting, might be seen as somewhat limiting. It would be intriguing to explore how learning theories could benefit patients, but Rotthoff doesn't address this.   Thank you for this comment. From the elaborated and disclosed lines of thought, patients can also benefit, provided these thoughts are translated into patient-friendly language and discussed with the patient in a dialogical manner. I have emphasized this more strongly   

 I'd like to delve into some of the tips that, in my view, require further clarification or elaboration.

- Tip 1: Consider the Role of Emotions in Learning. Rotthoff repeatedly refers to a review by Tyng, which is a general psychological review not specifically focused on early-stage medical students. He doesn't touch on, for instance, McConnell's work (AM 2016) Impact of Emotions on Learners," nor does he address the emotions experienced by patients in clinical encounters, an aspect often overlooked by medical professionals. Teacher emotions also go unmentioned.

Many thanks for the reference. I added insights from the work of McConnell and Eva and also referred to the influence of emotional experiences during bedside teaching on the patient. Tip 2: Begin with a Debriefing. This is just one part of the initial steps in bedside teaching. Both Ramani and Carlos place debriefing in a broader context that includes the patient and emphasizes creating a safe learning environment. Rotthoff's references are primarily related to simulation settings, which, while important, might not align with the primary focus of bedside teaching, in my opinion. However, a safe learning environment remains essential in every setting. Thank you for your comment and I completely agree. To be more specific, I would like to distinguish the prebriefing from briefing. Prebriefing essentially serves the more general preparation of the lesson for student, patients, and teachers. It includes, for example, informing the patient and seeking his consent. The classic briefing that I am addressing here refers to the immediate start of the bedside teaching. I agree that the learning atmosphere is also highly relevant, and I have emphasized its importance. Despite frequent mentions of briefings in academic literature, there is a notable lack of evidence regarding the effect on actual workplace learning. Although simulation training differs from real workplace experience, principles of some learning theories seem to be applicable to real clinical situations, particularly when compared to complex simulation scenarios. I have tried to work out this more strongly. Tip 4: Utilize Visual Thinking Strategies. This tip finds support in a systematic review by Cerqueira, which cautiously concludes that the VTS approach can foster essential clinical competencies and encourage more in-depth visual analysis when observing a patient. Hailey et al.'s chapter discusses two studies that demonstrate an impact on healthcare education. Both do not mention bedside teaching. I agree and, according to my own literature searches, there is no empirical evidence regarding transfer VTS to direct bedside teaching. Like the others, this tip is derived from learning theory considerations. I have therefore revised this tip as more relative in terms of language.

- Tip 5: Develop Epistemological Beliefs Alongside the Biopsychosocial Model. This tip requires further clarification, especially in the context of bedside teaching. Most clinicians may not fully grasp this theory, let alone be able to address it in a meaningful way. Rotthoff doesn't mention continuous professional development for clinical teachers, and, in my humble opinion, his expectations of the average clinician involved in bedside teaching might be a little too high.

I am aware that the theory of epistemic beliefs is not yet so strongly anchored in the medical context, but I consider it to be very relevant and useful. I have therefore revised this section considerably and hopefully made it clearer and easier to understand by examples.

- Tip 6: Set Priorities in the Patient Encounter. This is part of the preparation process advocated by Ramani and Carlos, but it's more comprehensive.

This certainly includes an aspect of the prebriefing, but at the same time, it aims to raise awareness about the need for content reduction during the actual bedside teaching. It also seeks to explore the didactic possibilities. For instance, as suggested, the patient perspective can be more strongly incorporated. Additionally, I have refined the theoretical learning background in this context.

  

- Tip 8: Think Aloud. The current support from the literature for thinking aloud in bedside teaching seems to lack weight.

I have revised this section, and these suggestions too are derived from learning theory and should not reflect the state of empirical research (which has also been largely lacking to date). I hope this has now become clearer with the revision.

- Tip 9: Use Socratic Questioning. Similar to tip 5, this also requires training for clinicians. If not executed correctly, Socratic questioning may be perceived as "pimping," embarrassing learners and reinforcing the teacher's position of power over the team, potentially having the opposite effect.

I agree that this questioning technique can also be counterproductive if it is not used in a good learning atmosphere and in a way that is comprehensible to the students and also with regard to the patient's condition. I have now gone into the underlying conditions in more detail in the text. 

- Tip 10: Perform Evidence-Based Clinical Examination. In my opinion, this tip stands out when discussing cognitive theories as the foundation for bedside teaching.

Self-critically, I have to confirm and therefore deleted this tip.

  

- Tip 11: Conclude with a Debriefing and Feedback. This insight isn't novel and has been mentioned by numerous authors, including Ramani and Carlos, who place it in a broader context that includes the patient.

Certainly, debriefing is not a novel concept and has been frequently discussed. Building on the commentary for tip 2 "Briefing," the aim of this tip is to derive and work out why which steps in the debriefing can have a potential benefit. I have revised the text accordingly.

   The conclusion section is formulated cautiously and serves more as a summary than a true conclusion. A conclusion should build upon the information presented in the rest of the article, and including a reference in it does not strengthen the conclusion. I have thoroughly revised the conclusion

 In summary, I genuinely appreciate Rotthoff's novel approach to improving bedside teaching. However, the current draft of this personal perspective on how to enhance bedside teaching lacks the substantiation and scientific rigor necessary to implement the suggested tips and truly enhance bedside teaching. Thank you! Surprisingly, there is a lack of scientific empirical evidence for bedside teaching. To derive concrete suggestions for teaching and learning on bedside teaching from learning theories can offer new approaches for scientific evaluation.

# Reviewer report: Reviewer response for version 1

**DOI:** 10.21956/mep.21242.r35043

**Published:** 2023-10-24

**Authors:** Subha Ramani

**Affiliations:** 1 Brigham and Women's Hospital, Harvard Medical School, Boston, USA

The topic of bedside teaching remains ever more relevant in the digital age of healthcare and clinical teaching. I like the application of educational theories to the tips for bedside teaching, which adds something new to the field. Application of theoretical principles to practice is also clearly outlined as clinical teachers want practical strategies to apply at the bedside as time to teach shrinks.

 The manuscript is well written and well referenced.

 I do have some constructive comments:

1. The statement - integrate the different professional roles and competencies of doctors and medical students with one another - could use greater clarity. Integration of what competencies?
2. There is a body of bedside teaching literature that is missing - Ende, ten Cate, Verghese, Fletcher, Ramani. Though these authors have focused on the practical, they all emphasize the 'why' especially as related to humanistic values and other clinical skills that can only be taught at the bedside. Dornan's ExBL AMEE guide is also important to reference, only his BEME guide is listed. There is an older series by Ken Cox which is worth referring to.
3. Some topics are referenced heavily, others are not. That is fine for the most part. However, if an argument is made for each tip referring to application of theory, I might expect that link between theory and practice. Some tips are more theoretical and some are more practical. Is it possible to move from theory to practice within many of the tips?
4. I believe clinical reasoning may be a better term than critical reasoning for this topic.
5. If references are used within a tip, I might suggest balancing citations to include theoretical and practical so that forms a great link. As an example, under briefing only one reference to a simulation-based briefing is cited. Simulation is still not bedside. Many older articles about bedside teaching already emphasize briefing.
6. For visual thinking strategies, there are experts in use of art and humanities who have written a lot about its use in promoting human values. something to consider.
7. Most importantly, I am not seeing an emphasis on putting the patient at the heart of bedside teaching, engaging them at the bedside. Time as a barrier is very real and efficiency at teaching is also important to indicate.

This is a paper I enjoyed reading and many clinical educators will find useful. The constructive comments are aimed at further enhancing its applicability by clinical teachers worldwide, especially a smooth transition from theory to practice under each tip.

 I anticipate only minor additions or edits, no major ones are required. In its current form, I find the manuscript very useful.

If evidence from practice is presented, are all the underlying source data available to ensure full reproducibility?

Not applicable

Is the topic of the practical tips discussed accurately in the context of the current literature

Yes

Are all factual statements correct and adequately supported by citations?

Yes

Are the conclusions drawn balanced and justified on the basis of the presented arguments?

Yes

Are arguments sufficiently supported by evidence from the published literature and/or the authors’ practice?

Yes

Reviewer Expertise:

Clinical teaching, feedback, inclusion in education, mentorship

I confirm that I have read this submission and believe that I have an appropriate level of expertise to confirm that it is of an acceptable scientific standard, however I have significant reservations, as outlined above.

# Response

**Published:** 2024-03-01

**Authors:** Thomas Rotthoff

**Affiliations:** Medical Didactics and Education Research (DEMEDA), Augsburg University, Augsburg, Bavaria, Germany

The topic of bedside teaching remains ever more relevant in the digital age of healthcare and clinical teaching. I like the application of educational theories to the tips for bedside teaching, which adds something new to the field. Application of theoretical principles to practice is also clearly outlined as clinical teachers want practical strategies to apply at the bedside as time to teach shrinks.

 The manuscript is well written and well referenced. Thank you very much!

1. The statement - integrate the different professional roles and competencies of doctors and medical students with one another - could use greater clarity. Integration of what competencies?

To make it clearer, I have condensed the statements into the first sentence 

1. There is a body of bedside teaching literature that is missing - Ende, ten Cate, Verghese, Fletcher, Ramani. Though these authors have focused on the practical, they all emphasize the 'why' especially as related to humanistic values and other clinical skills that can only be taught at the bedside. Dornan's ExBL AMEE guide is also important to reference, only his BEME guide is listed. There is an older series by Ken Cox which is worth referring to.

Thank you for your note! Indeed, this is correct. In light of your recommendations, I have referred to Verghese and the AMEE Guide on Experiential Learning by Yardley, Teunissen, and Dornan in the introduction.

1. Some topics are referenced heavily, others are not. That is fine for the most part. However, if an argument is made for each tip referring to application of theory, I might expect that link between theory and practice. Some tips are more theoretical and some are more practical. Is it possible to move from theory to practice within many of the tips?

I have revised all the tips more closely aligned with learning theory backgrounds to get them more homogeneously. I introduced the theoretical background to each tip with a more detailed explanation.

1. I believe clinical reasoning may be a better term than critical reasoning for this topic.

I have changed this accordingly. 

1. If references are used within a tip, I might suggest balancing citations to include theoretical and practical so that forms a great link. As an example, under briefing only one reference to a simulation-based briefing is cited. Simulation is still not bedside. Many older articles about bedside teaching already emphasize briefing.

I agree, of course, that the briefing is already mentioned in previous articles. It was/is not my intention to present briefing as a new method but rather derive explanations from learning theory as to how the briefing could be meaningfully implemented. I have commented on this accordingly in the text to prevent any misunderstanding (look at tip 2)

1. For visual thinking strategies, there are experts in use of art and humanities who have written a lot about its use in promoting human values. something to consider.

Thank you for this note. I have now put the topic into context of art and humanities in more detail. (look at tip 4)

1. Most importantly, I am not seeing an emphasis on putting the patient at the heart of bedside teaching, engaging them at the bedside. Time as a barrier is very real and efficiency at teaching is also important to indicate.

I appreciate your criticism! I attempted to defuse that. The aim of the work at hand should not provide a general statement on bedside teaching, but rather to offer practical tips for a best possible implementation based on learning theory. 

 This is a paper I enjoyed reading and many clinical educators will find useful. The constructive comments are aimed at further enhancing its applicability by clinical teachers worldwide, especially a smooth transition from theory to practice under each tip.

 I anticipate only minor additions or edits, no major ones are required. In its current form, I find the manuscript very useful.
